# Neonatal Pain in Very Preterm Infants: Long-Term Effects on Brain, Neurodevelopment and Pain Reactivity

**DOI:** 10.5041/RMMJ.10132

**Published:** 2013-10-29

**Authors:** Ruth Eckstein Grunau

**Affiliations:** Department of Pediatrics, University of British Columbia and Child & Family Research Institute, Vancouver, Canada; and School of Nursing and Midwifery, Queen’s University Belfast, UK

**Keywords:** Behavior, brain, cortisol, development, pain, preterm, stress

## Abstract

Effects of early life psychosocial adversity have received a great deal of attention, such as maternal separation in experimental animal models and abuse/neglect in young humans. More recently, long-term effects of the physical stress of repetitive procedural pain have begun to be addressed in infants hospitalized in neonatal intensive care. Preterm infants are more sensitive to pain and stress, which cannot be distinguished in neonates. The focus of this review is clinical studies of long-term effects of repeated procedural pain-related stress in the neonatal intensive care unit (NICU) in relation to brain development, neurodevelopment, programming of stress systems, and later pain sensitivity in infants born very preterm (24–32 weeks’ gestational age). Neonatal pain exposure has been quantified as the number of invasive and/or skin-breaking procedures during hospitalization in the NICU. Emerging studies provide convincing clinical evidence for an adverse impact of neonatal pain/stress in infants at a time of physiological immaturity, rapidly developing brain microstructure and networks, as well as programming of the hypothalamic-pituitary-adrenal axis. Currently it appears that early pain/stress may influence the developing brain and thereby neurodevelopment and stress-sensitive behaviors, particularly in the most immature neonates. However, there is no evidence for greater prevalence of pain syndromes compared to children and adults born healthy at full term. In addressing associations between pain/stress and outcomes, careful consideration of confounding clinical factors related to prematurity is essential. The need for pain management for humanitarian care is widely advocated. Non-pharmacological interventions to help parents reduce their infant’s stress may be brain-protective.

## INTRODUCTION

The importance of pain in hospitalized newborns was first recognized in the 1980s. Prior to this time it was assumed that infants could not perceive pain early in life and that risks of pharmacological agents outweighed potential benefits. There were a series of seminal studies that began to define the field of infant pain. Concurrently, concerns about the developmental needs of very preterm neonates were raised.^1^ Routine endotracheal suctioning was found to initiate changes in cerebral blood flow, demonstrating that procedural stress in the preterm infant undergoing neonatal intensive care unit (NICU) care might affect the brain.^2^ In 1987, a landmark study found that neonates given anesthesia for surgery (rather than paralytics alone) showed better survival and fewer short-term morbidities.^3^ The first reliable validated infant behavioral pain measure was developed,^4^ that later became incorporated into multidimensional scales. Furthermore, at this time, rodent studies undertaken to address developmental neurobiology of pain began to reveal the biological underpinnings of pain early in life, especially the lower threshold in the neurologically immature organism, the key phenomenon of sensitization, and later maturation of descending modulation of nociceptive input.^5^,^6^ Understanding of infant pain and effects of pain exposure during weeks to months of hospitalization of infants born very prematurely has progressed greatly in the past 25 years; however, major gaps remain.

Evidence will be presented for long-term associations between repeated pain in the NICU in infants born very preterm (born ≤32 weeks’ gestation) and altered brain development, neurodevelopment, programming of stress systems, and later pain perception in infants born preterm. Given the extensive animal literature that has established mechanistic foundations for the impact of early environmental stress on the developing organism, together with the accumulating clinical evidence, it appears possible that exposure to prolonged and repetitive pain-related stress in infants born very preterm may potentially have long-term effects contributing to altered neurobehavioral development in vulnerable infants.

## DEVELOPMENTAL NEUROBIOLOGY OF PAIN

During weeks to months in the NICU, very preterm infants are exposed to a high number of life-saving skin-breaking procedures and interventions, as well as routine handling that elicit behavioral, physiological, and hormonal responses. The immature peripheral and central nervous system of the very preterm infant responds differently to pain.^6^ Pain and stress cannot be readily distinguished with pain assessment tools,^7^ thus here the term “pain/stress” will be used. Preterm neonates are more sensitive to pain/stress than infants born at full term. Preterm infants display a lower threshold to touch and more pronounced reflex responses to touch, compared to full-term infants.^6^ With repeated touch, this lower threshold declines further due to excitability of sensory neurons in the spinal cord. Due to lower touch threshold and sensitization, acute pain/stress reactivity in very preterm neonates varies depending on preceding interventions in the last hour,^8^ 24 hours,^9^ or cumulatively since birth.^10^,^11^ Specifically, these tiny neonates can respond to routine handling similarly to an invasive procedure; for example, a diaper change can elicit pain-like behaviors and physiologic responses if preceded by heel lance 30 minutes before.^12^ This phenomenon of sensitization thus is one mechanism whereby repeated pain/stress may become ongoing discomfort in the NICU. Furthermore, descending endogenous modulation of pain is not yet mature in very preterm infants,^6^ thus excitation elicited by pain/stress is not self-regulated during this phase of central nervous system (CNS) development.

Repeated pain/stress exposure in very preterm infants takes place at a time of rapid brain development and programming of the hypothalamic-pituitary-adrenal (HPA) axis. Synaptic connections are being formed, activity-dependent selective cell death (apoptosis) shapes the developing brain, and integrated cortical networks are becoming established.^13^ These processes are affected by “developmentally unexpected” stimulation.^1^ Moreover, electrophysiological evidence suggests that acute pain induces diffuse brain activation across multiple regions in preterm neonates,^14^ thus these neurologically immature infants are the most susceptible to long-term effects of pain.

## NEONATAL PAIN AND THE PRETERM DEVELOPING BRAIN

In the late second and third trimesters of fetal life, the period when the very preterm neonate born at 24–32 weeks’ gestation is in the NICU, the developing brain undergoes major changes in cytoarchitecture and development of functional networks. During this lengthy period of hospitalization of neonates born extremely preterm (≤28 weeks’ gestation) brain development includes establishment and differentiation of subplate neurons, alignment, orientation and layering of cortical neurons, elaboration of dendrites and axons, formation of synapses, selective pruning of neuronal processes and synapses, and proliferation and differentiation of glial cells.^15^ Using advanced magnetic resonance imaging (MRI) it is well-established that structural and functional differences in brain development are evident in preterm infants early in life, extending to adulthood.^15^–^18^ The etiology of neurodevelopmental problems in preterm infants who escape major brain injury is linked to disturbances in the expected organizational events in brain development.^19^ Furthermore there is “selective vulnerability” of specific cell populations, particularly the pre-oligodendrocytes and the transient subplate neurons.^20^ Early lineage oligodendroglia are vulnerable to insults that do not affect mature myelin-forming oligodendrocytes. These selective cell vulnerabilities in the preterm brain are reflected in white matter injury and have been linked to hypotension, infections, and inflammation.^20^,^21^ Multifocal white matter injury is the characteristic brain injury pattern in premature neonates, identified on MRI in about one-third of preterm neonates, and associated with motor and cognitive problems.^21^ White matter injury is followed by diffusely abnormal microstructural and metabolic brain maturation as preterm newborns develop from early in life to term-equivalent age. Abnormalities in brain maturation persist through childhood and adolescence and are associated with adverse neurodevelopmental outcomes.^22^–^26^ Moreover, MRI studies now point to injury to gray matter structures, such as the thalamus, cortex, and cerebellum, in the preterm brain.^27^ Subplate neurons, a transient cell population important for developing thalamocortical connections, are also vulnerable.^28^ Thalamocortical connections are disrupted in preterm infants,^29^ and altered functional connectivity in children and adolescents born preterm is an important risk factor for adverse cognitive outcomes.^25^,^30^ Importantly, there is altered cortical activation and functional connectivity during language and visual spatial processing in children and adults born preterm who have normal intelligence.^30^–^33^

Procedural pain/stress in very preterm infants is associated with abnormal brain development in the NICU, above and beyond other clinical risk factors associated with prematurity.^34^,^35^ These findings are consistent with animal studies revealing that inflammatory pain or acute pain from repeated injections increased apoptosis in the neonatal rat brain.^36^,^37^ Altered microstructure may be related to pain-related increases in proinflammatory cytokines in the periphery and the central nervous system, or over-stimulation of immature neurons.^35^,^38^,^39^ Pain-related stress may also have indirect effects on the brain, or may interact with other factors implicated in development, since our group found that greater neonatal pain/stress exposure (adjusted for clinical confounders) is associated with slower body and head growth in preterm infants from early in life to term-equivalent age,^40^ and on diffusion tensor imaging slower growth was associated with altered cortical gray matter in infants born very preterm.^41^ Mechanisms whereby pain-related stress exposure may affect multiple systems remain to be addressed.

Diffusely abnormal microstructure and metabolism^42^ and altered functional connectivity relative to term controls^29^ are associated with adverse neurodevelopment.^22^–^28^,^30^,^41^,^43^ Rodent studies provide strong evidence that early life experience can alter both the structure and function of the developing brain.^44^ In humans, exposure to stressors in the NICU is associated with regional alterations in brain structure and function. In two independent cohorts, Grunau, Miller, and colleagues found that greater neonatal procedural pain/stress (adjusted for clinical confounders including gestational age (GA), early illness severity, infection, surgeries, and duration of mechanical ventilation) is associated with altered brain development of preterm infants in the neonatal period^35^,^45^ and at school-age.^31^,^46^,^47^ We also showed that neonatal pain/stress is associated at age 7 years with altered IQ that is mediated by brain microstructural changes.^46^ Others found that neonatal brain maturation on MRI is improved (compared to standard care)by an intervention designed to help parents recognize and respond to stress in their preterm infant in the NICU.^48^ This parent stress-reduction intervention shows that effects of reduced neonatal stress can be detected on brain images with advanced MRI techniques.

Several mechanisms or interactions of different systems may potentially link neonatal pain-related stress exposure to altered neurodevelopment in this vulnerable population. The developing brain may be directly influenced via hemodynamic changes at a time of very immature autoregulation of cerebral blood flow, or indirectly through altered development of sleep/wake state architecture and programming of stress systems. Importantly, immature neurons are more sensitive to neurotoxic environmental influences.^13^ Pain in rat pups has been found to impact brain development adversely.^37^,^49^ However, until recently, relationships between pain and brain development in preterm infants were speculative.

Recently Grunau, Miller, and colleagues specifically addressed in preterm infants whether neonatal procedural pain/stress impacts the developing brain. In a longitudinal study, infants born very preterm at 24–32 weeks’ gestation underwent advanced MRI brain imaging early in their NICU stay and again at term-equivalent age.^35^ Higher pain-related stress quantified as the number of skin-breaking procedures (including tube insertions) from birth to term-equivalent age was associated with poorer neonatal brain development, after adjusting for multiple clinical confounding factors such as GA at birth, duration of mechanical ventilation, confirmed infections, surgeries, analgesia, and sedation exposure. Greater exposure to procedural pain-related stress was associated with reduced development of white matter (indexed by fractional anisotropy (FA)) and subcortical gray matter (measured by N-acetylaspartate-to-choline ratio (NAA/choline)—a marker of metabolism and density). Reduced FA was predicted by early pain prior to the first brain scan, whereas lower NAA/choline was predicted by pain exposure throughout the neonatal course. This pattern of results suggested a primary and early effect on subcortical structures with secondary white matter changes.

The potential for procedural stress in the NICU to affect the brain adversely was demonstrated long ago, in a study reporting that endotracheal suctioning altered neonatal cerebral blood flow.^2^ Recently, using electroencephalography (EEG) to measure electrical activity or near-infrared spectroscopy (NIRS) to examine cerebral blood flow changes, studies of cortical activity during procedures in the NICU have shown that procedures evoke responses in the cerebral cortex.^14^,^38^,^39^,^50^,^51^ Important differences in cortical response to touch and pain in preterm infants are evident in preterm compared to full-term neonates. In preterms, non-specific neuronal bursts of EEG activity widely dispersed in the brain were observed rather than a localized somatosensory response displayed by full-term infants.^14^ The findings of this study suggest a widespread immature EEG response, confirming that the preterm neonatal brain is more sensitive, consistent with poor capacity to distinguish tactile from nociceptive stimulation. This study therefore substantiated differential vulnerability of preterm infants to procedural pain higher in the central nervous system, since both non-painful mechanical as well as nociceptive stimulation evoked responses. Thus preterm infants appear to be potentially more vulnerable to repeated procedural pain/stress, due to immature capacity to differentiate nociceptive from tactile input. Together with low tactile threshold and sensitization to repeated touch in preterm neonates, the finding that evoked responses were widespread across the brain coalesces with other studies that have found that diaper change can induce as much biobehavioral response as blood collection under certain conditions.^52^

Stress of handling and procedures in the NICU is associated with changes in brain structure and function.^1^,^34^,^48^ There appears to be tremendous capacity for studies combining behavioral and physiological measures concurrently with EEG or NIRS, to address the impact of procedures in a multidimensional pain response reflecting many levels of the CNS.

At school-age, there appears to be only one study that has examined brain reactivity to painful stimuli in children born preterm. On functional magnetic resonance imaging (fMRI) at age 9–14 years, children born preterm displayed greater activation in the somatosensory cortex and other brain regions, compared to children born full-term with or without early hospitalization.^53^ Research to address understanding the effects of neonatal pain in very preterm infants as well as other infants exposed to NICU care, on responses in the brain evoked by touch and pain later in childhood and adolescence, is likely to receive a lot more attention in the future. As well, relationships between brain activation and self-report of pain need to be evaluated.

Importantly, programs designed to recognize infant stress cues and provide supportive care, compared to standard practice, are associated with improved brain maturation. The Neonatal Individualized Developmental Care and Assessment Program (NIDCAP), compared to standard care of preterm neonates, led to more mature coherence between frontal and other brain regions on EEG and better neurobehavioral function.^1^ Parent training in the NICU to help reduce stress in their very preterm infant was associated with better cerebral white matter microstructure, maturation, and connectivity on MRI at term-equivalent age,^48^ and with increased frontal EEG brain activity during sleep,^54^ compared to infants that received standard care.

In a longitudinal cohort of preterm infants followed from birth to school-age, Grunau, Ribary, and colleagues examined whether neonatal pain is associated with functional brain activity later in childhood. They found that greater cumulative neonatal pain-related stress was associated with altered spontaneous oscillatory brain activity (indexed as the ratio of gamma to alpha activity using magnetoencephalography) at age 7 years.^31^ Importantly, this measure of cortical activity was correlated with visual problem-solving abilities. We found that neonatal pain exposure (adjusted for clinical confounders) was associated with resting brain function in children born extremely preterm at 24–28 weeks, but not preterms born relatively more mature at 29–32 weeks. On EEG, progressive changes in the maturation of oscillatory brain activity are seen during the preterm period.^55^ The association between neonatal pain and brain activity only in infants born 24–28 weeks, but not later, may reflect the specific phases of development in thalamocortical systems during 24–28 compared to 29–32-week gestation periods.^56^ There is now evidence that the developing brain may be sensitive to procedural perturbations during a “critical window” in early life, suggesting that the long-term effects of pain are greatest prior to full-term birth. Studies revealing the widespread brain reactivity to a procedural intervention,^14^ as well as associations between pain and brain development,^35^ begin to address the role that pain-related stress might play in contributing to altered spontaneous neuromagnetic activity and atypical long-range task-dependent magnetoencephalographic synchronization,^33^ as well as perhaps the atypicalities in brain structure, function, and connectivity^16^,^57^ seen on MR and fMRI in children born very preterm.

While there is now initial evidence for both direct and indirect relationships between repeated prolonged exposure to neonatal procedural pain and the developing brain, a great deal more research is needed to reveal the mechanisms and relationships with other risk factors of prematurity.

## NEONATAL PAIN AND NEURODEVELOPMENT

While cerebral palsy has decreased among preterm infants in recent years,^58^ developmental motor co-ordination in the absence of other major impairments is highly prevalent.^59^ Moreover, cognitive problems remain common and may be increasing.^60^ Difficulties in attention, executive functions, cognition, language, visual-motor abilities, as well as behavior problems affect academic performance in children born very preterm,^61^ and persist to adulthood.^62^,^63^ Risk factors for poor neurodevelopment include many aspects of prematurity and the NICU experience, including gestational age below 29 weeks, lengthy mechanical ventilation, chronic lung disease, and infections.^64^ However, over and above key perinatal and neonatal clinical factors, higher pain exposure (operationalized as the number of skin-breaking procedures from birth to term-equivalent age) is independently associated with motor and cognitive development at 8 and 18 months’ corrected age (CA),^65^ IQ at age 7 years,^46^ and internalizing (depressive, anxiety, somatic symptom) behaviors at 18 months^66^ and at school-age.^67^ Importantly, the relationship between neonatal pain and neurodevelopmental outcomes appears to be mediated by altered brain maturation.^35^,^67^ Visual-motor and fine and gross motor problems, in the absence of other disabilities—defined as developmental co-ordination disorder (DCD)—are particularly common in children born very preterm.^59^ Our work has recently provided the first evidence that repeated neonatal pain-related stress contributes to changes in the neonatal corticospinal tract (independent of clinical confounders) and thereby motor functions at 18 months’ CA.^45^ Visual-spatial memory problems are also highly prevalent among preterms and appear to be related to altered functional brain activity, characterized by higher ratio of gamma to alpha oscillations.^31^

Early pain-related stress may affect specific developmental domains via different systems. As described above, pain appears to affect cognition and motor function through changes to brain microstructure and function. In contrast, internalizing behaviors that include depressive, anxiety, and somatic symptoms—all stress-sensitive—may be more related to altered programming of the hypothalamic-pituitary-adrenocortical (HPA) axis. This distinction is somewhat arbitrary, however, given that cortisol levels are also involved in brain function. At 18 months’ CA, we found that cortisol levels were altered across the first two years of life in extremely preterm infants.^68^,^69^

Relationships between physiological and behavioral reactivity to external stimulation such as touch or pain, the contribution of concurrent clinical events in the NICU such as hypotension, infection, and inflammation, and how these may interact to affect mechanisms underlying motor, cognitive, and complex behavioral development will require relevant animal models integrated with clinical research.

## PAIN, SLEEP, AND BRAIN DEVELOPMENT

Sleep architecture and sleep–wake states start to develop during the third trimester of fetal life. Sleep has an important role in brain development, and disturbances in sleep–wake patterns affect the developing central nervous system.^70^,^71^ It is well-documented that routine procedures in the NICU such as blood collection impact the sleep–waking state.^72^ Shifts in sleep–wake state are an intrinsic part of infant pain assessment. It is unclear to what extent repeated painful procedures may alter or disrupt development of normal sleep–waking state patterns. Moreover, opioids decrease rapid-eye-movement sleep, thereby affecting sleep structure in preterm neonates.^73^ Surprisingly, noxious-specific EEG potentials were found not to be sleep state-dependent, as the proportion of response for those who did and did not exhibit a noxious-specific somatosensory reactivity was the same in the awake infants compared to those who were sleeping.^14^ However, very preterm infants in the NICU typically are in a light sleep state, spending little time awake or in deep sleep. Despite the central role of sleep in relation to brain function, there is limited knowledge of the role of repetitive pain and handling on sleep disruption and development of brain maturation in this fragile population.

## PAIN AND THE HYPOTHALAMIC-PITUITARY-ADRENAL (HPA) AXIS

The adrenal cortex releases the primary stress hormone cortisol (corticosterone in rodents) that contributes to adaptation under different levels of stress and to changes in brain function. A major literature extending for more than 50 years has established long-term effects of early social adversity, extending to the transformative contributions of Meaney and colleagues on the mechanisms of biological encoding of maternal behavior (for a review see Champagne^74^). Early in life, environmental stress can lead to altered programming of the hypothalamic-pituitary-adrenal (HPA) axis—reflected in shifts in levels of corticosterone in rodents and cortisol as the main stress hormone in humans.^75^ Surprisingly, unlike effects of the stress of maternal separation, no changes in HPA activity were found in animal studies of long-term effects of early physical pain.^36^,^76^ Investigation of maternal behavior revealed that after a rat pup was exposed to pain, maternal licking and grooming increased, thereby preventing changes to stress hormone expression.^76^

Very preterm infants in the NICU undergo both the physical stress of repeated painful procedures and the concurrent social stress of maternal separation. While in the NICU, infant cortisol levels are often lower than expected, given the degree of stress, and are affected by multiple medical factors such as hypotension, infection, and inflammation,^77^ making it difficult to separate effects of pain from confounding current factors. Our work has revealed associations between cumulative procedural pain and altered “resting” (i.e. unstimulated) cortisol levels independent of clinical confounders while in the NICU,^11^ in infancy,^68^,^69^ and at school-age.^78^ However, the pattern is complex. Lower cortisol levels in the NICU and at 3 months switched to up-regulation (higher cortisol) at 8 and 18 months’ CA,^68^,^69^ then to lower than expected levels at school-age. This type of shift is seen in other highly stressed populations, and either too high or too low cortisol levels potentially can impact brain function.^79^ Furthermore, we found that infant and toddler behaviors were related to the pattern of cortisol expression at 3, 6, 8, and 18 months,^80^–^83^ suggesting persistent alteration of stress system programming has functional relevance in these children. Research is needed to examine these shifting trajectories of cortisol expression in children born very preterm, how cortisol levels may interact with altered brain maturation, and the extent to which these changes may be downstream effects of pain in the NICU.

## STRESS, HPA AXIS, AND IMMUNE SYSTEM

There are complex bidirectional influences between the central nervous system, the HPA axis, and the immune system.^84^ Chronic activation of stress responses induces ongoing production of glucocorticoid hormones. In turn, glucocorticoid receptors are expressed on many types of immune cells, and bind cortisol, which may interfere with the function of the proinflammatory transcription factor NFκB that regulates the activity of cytokine-producing immune cells. Furthermore, changes in gene expression through effects of glucocorticoid hormones and catecholamines can dysregulate immune function. In general, studies of stress and immune function in humans have focused on psychological or social stressors. In contrast, physical stress of repetitive pain in neonates does not appear to have been addressed until recently. Grunau and colleagues^78^ found a sex-specific relationship between normal genetic variation of *NFκBIA* rs2233409, extent of procedural pain/stress exposure, and hair cortisol level (an index of cumulative stress) at school-age in children born very preterm. The *NFκBIA* gene encodes IκBα, a critical negative regulator of the transcription factor NFκB.^85^ In preterm boys but not girls with the *NFκBIA* rs2233409 minor allele (CT or TT), greater neonatal pain-related stress (number of skin-breaking procedures from birth to term), independent of medical confounders, was associated with lower hair cortisol at age 7 years. Moreover, the minor allele of *NFκBIA* rs2233409 was associated with higher secretion of inflammatory cytokines, suggesting that neonatal pain/stress may act as a proinflammatory stimulus that induces long-term immune cell activation. These findings are the first evidence that a long-term association between early pain-related stress and cortisol may be mediated by a genetic variant that regulates the activity of NFκB, suggesting possible involvement of stress/inflammatory mechanisms in HPA programming, at least in boys born very preterm.

## PAIN THRESHOLD IN PRETERM INFANTS AFTER NICU DISCHARGE

Early studies used a parent questionnaire to measure pain sensitivity in preterm children. Parents reported lower pain sensitivity to everyday bumps, scrapes, and falls in micropremies born at or below 800 g compared to control children born full-term.^86^ At 8–10 years of age, rather than parent report, children rated pictures depicting pain in medical, recreational, and daily living settings, and preterms born less than 1001 g were compared to controls.^87^ While overall ratings were similar to age-matched peers born full-term, the children born extremely preterm rated medical pain intensity significantly higher than psychosocial pain, unlike the control group. Child IQ and maternal education were statistically adjusted in comparisons between the two groups. Duration of time in the neonatal intensive care unit among the preterm children was related to higher ratings in pain affect in recreational and daily living settings.

Studies that have directly compared behavioral and physiological pain responses in former preterm compared to full-term infants long after NICU discharge revealed that age at testing is important. At 4 months’ CA, i.e. age adjusted for prematurity, infants born at or below 800 g (i.e. micropremies born below 26 weeks’ gestation) showed no differences in behavioral or cardiac responses to blood collection by finger lance, a site that had not been previously used and thus had no peripheral pain stimulation in the past.^88^ This finding was very unexpected given the prolonged pain/stress exposure in the NICU in infants born so early. In the same cohort at 8 months’ CA, the preterm infants displayed a greater facial pain response to a finger lance in the first few seconds, and more rapid dampening of behavior and heart rate, compared to full-term infants.^89^ These findings of differences in responses emerging over time rather than disappearing appear to be consistent with rodent studies.^90^ Since the finger lance may have been too minor to elicit differences between the preterm and full-term children, we undertook a study of reactivity to immunization injections at 4 months’ CA in infants born at or below 32 weeks’ gestation, compared to full-term controls.^91^ Again, there were no significant differences in facial or cardiac responses. However, sex differences were evident in cortisol response to immunization, with preterm boys displaying a lower cortisol response, although facial behavior and heart rate reactivity did not differ between boys or girls.

Later in childhood, there have been a number of experimental studies of pain threshold in children born preterm, revealing complex effects. Adolescents born preterm had more tender points and lower pain threshold compared to their term-born peers.^92^ In school-age children born preterm, using quantitative sensory testing, both hypersensitivity and hyposensitivity to pain have been found, compared to children born full-term, depending on the type of pain stimulus and duration.^93^,^94^ Increased sensitivity to brief heat and reduced sensitivity to prolonged heat were found at sites that were not injured in infancy. These findings are consistent with studies of long-term effects of early pain in rat pups.^90^ Importantly, neonatal surgery accounted for differences in pain sensitivity in children born at or below 25 weeks’ gestation.^94^ Given the extent of pain exposure in infants born that early, the minimal difference in pain sensitivity between micropremies who had not undergone surgery and controls was very surprising and re-assuring.

In some other studies of long-term changes in pain sensitivity following early surgery, both preterm and full-term children have been included in samples. Pain threshold at school-age depended on type of surgery and whether threshold was tested in the region of surgery. For example, sensitivity among children who had chest surgery in infancy showed reduced sensitivity to touch, cold, and heat in the region of the surgery.^95^ In other studies, increased sensitivity was evident later in young children with a history of surgery.^96^,^97^ An important finding was the need for more intraoperative anesthesia and more postoperative analgesia in children who had surgery previously, compared to children having their first surgery. In contrast, toddlers who had surgery did not differ in their behavior or physiological responses to immunization.^98^

Pain threshold may be altered in children long after early surgery, especially in regions of prior tissue damage, which is important if another surgery is needed later. However, the direction and magnitude appear to depend on many factors that are unclear at this point, perhaps at least in part due to inclusion of children born preterm and full-term. It is unknown whether physiological immaturity at the time of surgery may contribute to the long-term impact on later touch or pain threshold.

Although repeated procedural pain early in life in infants born preterm is associated with altered sensory thresholds when tested in experimental conditions, studies of self-report, however, suggest no differences in “everyday” pain or pain syndromes compared to full-term controls in adolescents or young adults born preterm.^63^,^92^,^94^,^95^,^99^,^100^ Furthermore, in a prospective cohort study of self-completed questionnaires in 18,572 participants at age 45 years, there was no significant association between adults born at low birth-weight or very low birth-weight and reports of chronic pain, with or without adjustment for medical and social confounders.^101^ Thus, although there is evidence that touch and pain thresholds differ, as well as altered brain activity in response to pain in children born preterm, the literature is consistent that there is no evidence for increased prevalence of pain syndromes in adulthood.

## PARENTING AND PAIN IN PRETERM INFANTS

In a longitudinal randomized trial where mothers were taught how to reduce stress in their low-birth-weight infants, IQ scores at 9 years of age were >10 points higher in the intervention group.^102^,^103^ In another trial, preterm infants exposed to a parental intervention similar to that used in the randomized trials cited above^102^,^103^ demonstrated enhanced brain maturation and connectivity on MRI at term-equivalent age.^48^ In a cohort study, we found that greater positive maternal interaction buffered the relationship of neonatal procedural pain exposure with poorer focused attention in very preterm infants at 8 months’ CA and was protective against internalizing (anxiety/depressive) behaviors at 18 months’ CA. Yet, it remains unknown whether the relation between adverse brain development and impaired cognitive outcome is improved by supportive parent–child interactions.

Potentially protective factors in the face of early biological adversity are recognized as a crucial focus to explain the wide variability of neurodevelopmental outcomes in very preterm infants.^104^ Importantly, there is converging evidence that preterm infants are more developmentally vulnerable to their parent interaction.^82^,^83^,^104^ These data indicate that parents’ behaviors play a key role in their child’s neurodevelopment and may compensate for adverse clinical exposure and compromised early brain development. Maternal care of rat pups positively affects adult sensitivity to pain following neonatal inflammation. Interestingly, in children born preterm, Hohmeister et al.^105^ reported altered pain behavior when the mother was present. The role of parenting, social modeling, and other environmental contextual influences on later pain threshold has received little study in children born preterm.

## CONCLUSION AND FUTURE DIRECTIONS

There is now convincing evidence that repeated neonatal procedural pain/stress in very preterm infants in the NICU may have the potential to adjust set points in biological circuits and alter brain microstructure and function, stress systems, neurodevelopment, and stress-sensitive behaviors. This suggests potential mechanisms that may contribute to the etiology of neurodevelopmental and behavioral problems in children born very preterm. Genetic variation contributing to diverse effects has just begun to be examined,^78^ and epigenetic changes are likely to provide mechanistic understanding of how early pain experience “gets under the skin.” Pain threshold appears to be changed in infants exposed to surgery, above and beyond routine procedural pain/stress. However, long-term effects of repetitive pain are complex. Surprisingly, the threshold differences seen in preterm children at school-age compared to full-term children are not accompanied by self-report of aberrant pain syndromes, despite different engagement of brain regions during functional brain imaging.

Addressing whether specific approaches to pain management in the NICU may improve the developing brain and promote better long-term outcomes is urgently needed. While morphine does not appear to affect developmental outcomes adversely, pre-emptive continuous morphine infusion for pain management has yielded little if any benefit for prevention of morbidities and is no longer recommended. The burgeoning field of pharmacogenomics in future holds promise for individualizing pharmacologic pain management but has not yet been addressed with preterm infants. Currently, sucrose is widely used for routine minor procedural pain; however, there is a dearth of research into whether there are long-term positive or negative effects of repeated sucrose exposure in tiny babies. Supportive “environmental care” and parent involvement show promise for reducing stress in preterm neonates, thereby improving brain structure and activity. The extent to which non-pharmacologic pain management may prevent long-term effects of neonatal pain remains unknown.

## Abbreviations:

CA: corrected age;
CNS: central nervous system;
DCD: developmental co-ordination disorder;
EEG: electroencephalography;
FA: fractional anisotropy;
GA: gestational age;
HPA: hypothalamic-pituitary-adrenal;
NAA/choline: N-acetylaspartate-to-choline ratio;
NICU: neonatal intensive care unit; NIDCAP, Neonatal Individualized Developmental Care and Assessment Program;
NIRS: near-infrared spectroscopy.
